# Circadian clocks of both plants and pollinators influence flower seeking behavior of the pollinator hawkmoth *Manduca sexta*

**DOI:** 10.1038/s41598-018-21251-x

**Published:** 2018-02-12

**Authors:** Myles P. Fenske, LeAnn P. Nguyen, Erin K. Horn, Jeffrey A. Riffell, Takato Imaizumi

**Affiliations:** Department of Biology, University of Washington, 24 Kincaid Hall, Box 351800, Seattle, WA 98195-1800 USA

## Abstract

Most plant-pollinator interactions occur during specific periods during the day. To facilitate these interactions, many flowers are known to display their attractive qualities, such as scent emission and petal opening, in a daily rhythmic fashion. However, less is known about how the internal timing mechanisms (the circadian clocks) of plants and animals influence their daily interactions. We examine the role of the circadian clock in modulating the interaction between *Petunia* and one of its pollinators, the hawkmoth *Manduca sexta*. We find that desynchronization of the *Petunia* circadian clock affects moth visitation preference for *Petunia* flowers. Similarly, moths with circadian time aligned to plants show stronger flower-foraging activities than moths that lack this alignment. Moth locomotor activity is circadian clock-regulated, although it is also strongly repressed by light. Moths show a time-dependent burst increase in flight activity during subjective night. In addition, moth antennal responsiveness to the floral scent compounds exhibits a 24-hour rhythm in both continuous light and dark conditions. This study highlights the importance of the circadian clocks in both plants and animals as a crucial factor in initiating specialized plant-pollinator relationships.

## Introduction

Plants and their pollinators provide one of the most studied examples of mutualistic interactions. To facilitate these interactions, many flowers produce and display phenotypes that operate as “advertisements” to the pollinators including shape, color^1^, scent^2^, number of open flowers^3^, etc. The evolution of these advertisements allows for some plant species to tune their relationship to a specific pollinator or set of pollinators, adopt a generalist approach to attracting pollinators, or a combination of both^4^. Experimental manipulation of floral traits has allowed researchers to examine the ease with which changes in floral advertisements can affect pollinator visitation. For instance, changes in floral color affect the preference of bees and hummingbirds for *Mimulus* flowers^5,6^, and also the preference of bees and hawkmoths for *Petunia* flowers^7^.

A critical aspect for selectively attracting certain pollinators is temporal control of the flower’s advertisements and resources. To facilitate this, many components of floral attraction oscillate on a daily schedule, including floral opening, scent emission^8^, nectar production^9^, flower orientation^10^, etc. Many pollinators are generally known to have temporal restrictions on their activity^11^, although few detailed time courses of pollinator behavior have been recorded^12,13^. These observations indicate the ecological and evolutionary significance of their internal timekeeper mechanism, the circadian clock. However, it has remained largely unknown whether and how the circadian clock in each plant and insect is involved in maintaining their mutually beneficial plant-pollinator interactions.

The hawkmoth *Manduca sexta* has a wide distribution across the Americas, where its larval form, the tobacco hornworm, is a well-known pest of Solanaceae crops^14^. The adult moth is generally known as a nocturnal and/or crepuscular nectivore of various plant species (often Solanaceae), and is a model organism in the fields of animal behavior, neuroscience, and insect flight^15,16^. *M. sexta* maintains a nocturnal relationship with the flowers of *Petunia axillaris* in the wilds of Uruguay^17^. Typical of nocturnal hawkmoth-pollinated species, *P. axillaris* has highly reflective white flowers with long narrow corolla tubes, and emits a robust bouquet of scent at night^18^. This nocturnal scent release involves compounds produced primarily from the Floral Volatile Benzenoid/Phenylpropanoid (FVBP) pathway, which is active during the evening^17^. The FVBP pathway has been studied extensively in the research model *Petunia hybrida* cv. Mitchell^19^, which has a nearly identical floral morphology and scent profile to its parent species *P. axillaris*. Evening-expressed transcriptional regulators, such as *ODORANT1* (*ODO1*), *EMISSION OF BENZENOIDS I* (*EOBI*), and *EOBII*, upregulate the transcription of enzymes involved in the processing of precursor molecules through the FVBP pathway^20–22^. The timing of daily scent emission is regulated by the circadian clock^19^. To restrict *Petunia*’s characteristic scent emission and its underlying FVBP metabolism to night, the morning-expressed clock component *Petunia hybrida* LATE ELONGATED HYPOCOTYL (PhLHY) directly represses the expression of *ODO1* and other key enzymatic genes during the daytime^23^. The influence of the *M. sexta* clock on this plant-pollinator relationship is less characterized. Circadian clock-dependent behavioral and physiological responses to pheromones and food-related odors were described in other insect species, especially *Drosophila melanogaster*^24–28^. In other insects, circadian modulation of both locomotor activity and antennal responsiveness to scents are likely mechanisms of rhythmic behavioral responses, though little evidence exists for circadian modulation of plant-pollinator interactions. Antenna from *M. sexta* exhibit a stronger response to scent collected from *P. axillaris* flowers during the night vs the day^17^, but no study to date in *M. sexta* has examined whether changes in antennal sensitivity to a standard quantity of scent changes over the course of 24 hours. In a laboratory setting, tobacco plants with genetically altered clocks showed a change in fitness compared to wild-type plants when exposed to *M. sexta* as a pollinator^29^.

To better understand the nocturnal attraction of *M. sexta* to *P. axillaris* flowers, we conducted a series of experiments. First, we experimentally disrupted the internal timing of either the plant or insect, to determine if their internal clocks influence the success of the interaction. Second, we exposed *M. sexta* to different light conditions in ordinary sets of circadian experiments that are designed to assess the effects of the circadian clock and light on the locomotor activity of *M. sexta*. We then analyzed whether the internal circadian time of *M. sexta* impacts its ability to respond both behaviorally and physiologically to floral scent. Here we provide evidence that the synchrony of plant and insect circadian rhythms is important, as well as additional evidence that the *M. sexta* clock modulates its half of this interaction by gating locomotor activity and floral odor sensitivity to the night.

## Results

### Circadian timing is important for floral visitation of *M. sexta*

**Figure 1:**
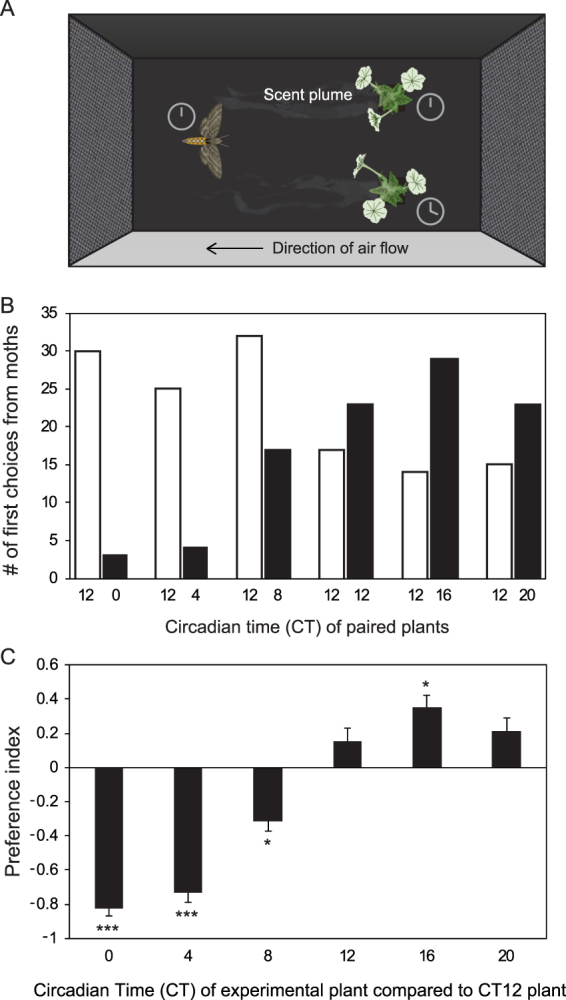
Synchronization of plant and pollinator clocks is important for floral visitation. (**A**) Diagram of experimental setup: an adult male *M. sexta* moth at Circadian time 12 (CT12) is given 5 minutes to make a first choice between two stimuli: the control plant (also at CT12) and an experimental plant entrained to another internal time (with a comparative control for CT12). Plants were randomly assorted. (**B**) Raw data from the choice experiment. Each pair indicates the circadian time of the two plants in the choice assay, a control at CT12 (shown in white), and an experimental plant at another time (shown in black). The number of moths shown is the sum of of moths recorded from independent replicate choice experiments. (**C**) Preference index of the choice experiment data. Each bar represents the preference for 30+ individuals (tested separately) at one timepoint comparison of the 24-hour experiment. Error bars represent the standard error of the binary distribution. Asterisks denote choices which are significantly different from random in a binomial exact test (*p < 0.5, ***p < 0.001). See Supplementary Fig. 1 for a full schematic of the wind tunnel apparatus.

Previously, we examined the role of the circadian clock in regulating the emission of floral scent from *P. hybrida* cv. Mitchell, a commercial species derived from *P. axillaris*, with which it shares similar phenotypes (white flowers, heavy emission of benzenoid volatiles at night). We found that the morning-expressed clock component PhLHY directly represses the expression of genes related to floral volatile synthesis during the daytime, restricting scent synthesis and emission to the night^23^. Nightly emission of scent could confer a fitness advantage to plants in part by restricting pollination to a subset of efficient pollinators. A plant’s temporal control of floral emission only describes one half of this nightly interaction, however; the pollinator must also play its part. To examine the importance of clock synchronization to successful floral visitation, we gave naïve male moths with an internal circadian time of 12 (CT12, evening) a choice for flower visitation between two flowering plants of wild-type *P. axillaris*. One choice was a flower which was also experiencing CT12, and the other was experiencing an alternate phase of the circadian clock to the moth clock (Fig. 1). Moth preference for plants decreased as the plant’s circadian time became earlier than CT12, with moths choosing CT12 plants over CT0 plants in ~90% of trials (PI = −0.82; binomial exact test, p-value = 1.4 × 10^−6^). Slightly greater preference was shown for plants experiencing CT16 over CT12, likely because CT16 is closer to the peak emission time of floral scent in *P. axillaris*^17^ (PI = 0.34; binomial exact test, p-value = 0.03). These results indicate that the internal circadian time information of plants may influence the success of the plant-pollinator interaction.

**Figure 2:**
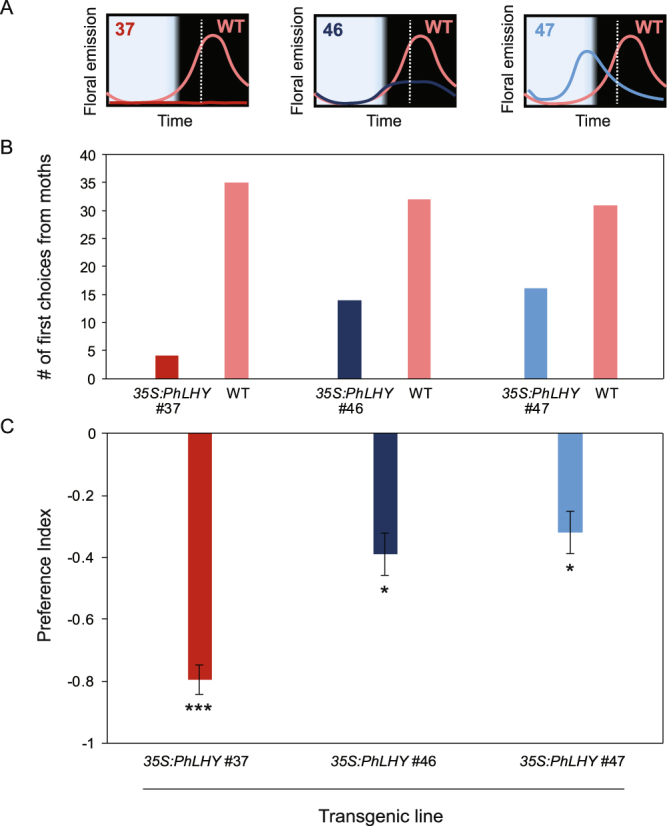
Transgenic *Petunia hybrida* with disrupted clocks receive reduced preference from *M. sexta* vs wild-type *P. hybrida*. Moths entrained to 12L:12D conditions were given a choice between 2 plants, a wild-type (WT) *P. hybrida* plant and a transgenic plant with altered clock rhythm: (*35 S:PhLHY* #37: arrhythmic, *35 S:PhLHY* #46: early phase shift, *35 S:PhLHY* #47: early phase shift). (**A**) Emission profiles of the plant lines for each experiment is shown above the corresponding bar (emission profiles synthesized from previously published data^23^. All organisms were entrained to 12L:12D conditions, and the choice experiment carried out at ZT16 (denoted as the white dotted line in the emission profile). (**B**) The raw data of each choice experiment between clock-altered line and WT *P. hybrida*. (**C**) Preference index of the choice experiment data. Each bar represents the preference for 39+ individuals (tested separately) for each choice experiment. Error bars represent the standard error of the binary distribution. Asterisks denote choices which are significantly different from random in a binomial exact test (*p < 0.5, ***p < 0.001). See Supplementary Fig. 1 for a full schematic of the wind tunnel apparatus.

Further evidence of the clock’s potential importance to floral visitation was gathered by examining pollinator choice among plants with genetically altered clocks (Fig. 2). In our previous work, a line of transgenic plants with arrhythmic clock functionality was produced by constitutive expression of the clock gene *PhLHY*. In this experiment, wild-type and transgenic plants were grown under the exact same light/dark conditions, but the timing of their internal clocks differed genetically. Plants with an arrhythmic clock (*35 S:PhLHY* #37) showed an almost complete absence of FVBP floral scent emissions^23^. Moths chose wild-type *P. hybrida* over *P. hybrida* with arrhythmic clocks (*35 S:PhLHY*) in 90% of trials (PI = −0.79; binomial exact test, p-value = 3.35 × 10^−7^) (Fig. 2B,C). Wild type plants also received more visits when competing against transgenic lines with shorter clock periods (*35 S:PhLHY* #46 and #47) (Fig. 2B,C). These results further indicate that aligning the internal time of plants and moths is important, as part of the clock’s role in synchronizing physiology with the surrounding environment.

**Figure 3:**
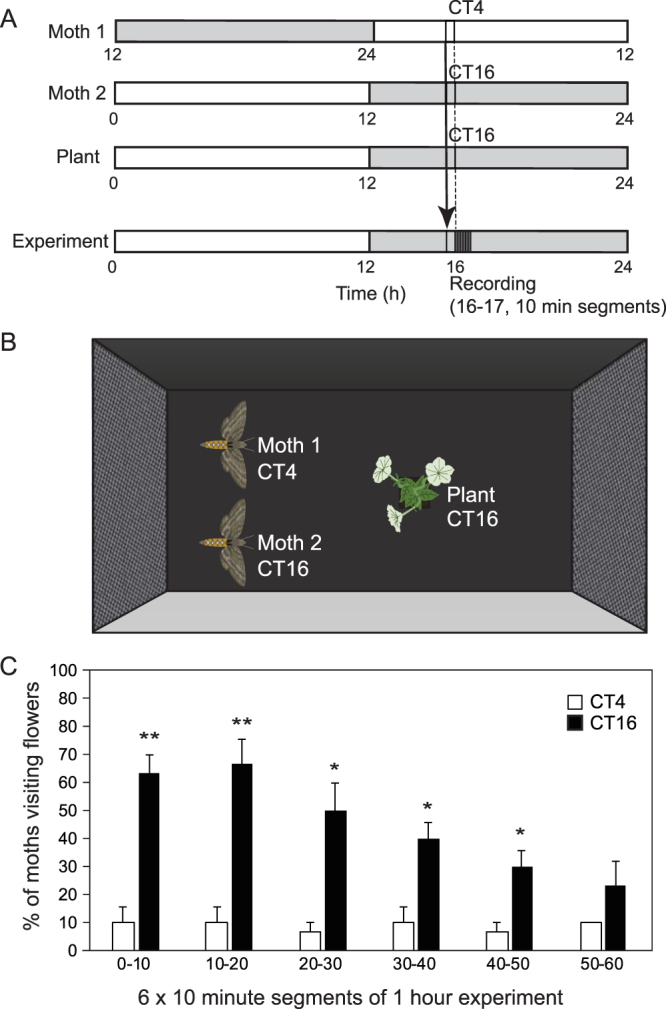
Circadian time of moths affects foraging activity on *P. hybrida* flowers. (**A**) Two groups of male *M. sexta* moths (Moth 1 and Moth 2 groups) entrained to different 12L:12D conditions offset by 12 hours; flowering *P. axillaris* (Plant) was also entrained to 12L:12D conditions. An arrow indicates that moths were transferred from separate environmental chambers to the experimental chamber 30 minutes prior to the experiment. Actual infrared recording occurred between ZT16 and ZT17 (indicated by the dark gray box). The number of moths visiting flowers was counted in each 10-minute window. (**B**) Diagram of experimental setup in wind tunnel chamber. 30 minutes prior to the 1-hour experiment, groups of CT4 and CT16 moths were simultaneously introduced to the wind tunnel, and the CT16 plant was introduced at the beginning of the experiment. (**C**) The 1-hour experiment was divided into six 10-minute segments, and in each segment the percentage of CT4 and CT16 moths that visited flowers is reported (total 10 moths each/experiment). The experiments were biologically repeated three times with independent samples. Asterisks denote the significance value assigned by t-test comparison of CT4 vs. CT16 numbers at each timepoint (**p-value < 0.01, *p-value < 0.05).

Next, we analyzed whether altering moths’ internal time affected this interaction. To test this, we split naïve moth populations into two groups and entrained them separately under different light/dark conditions. One group of moths was entrained to regular 12-hour light/12-hour dark conditions, which were the same light/dark conditions to which plants were entrained (although in separate chambers). The other group was entrained under reverse light/dark conditions, in which light conditions were 12 hours apart from the regular conditions (Fig. 3A). We introduced these two groups of moths together with scent emitting flowers at the early part of night (Fig. 3A). We waited 30 minutes to let moths adjust to the experimental conditions, and then started measuring the foraging activity of moths experiencing “daytime” (Moth 1 group, referred to as “CT4 moth”) and “nighttime” (Moth 2 group, “CT16 moth”) on a flowering *P*. *axillaris* plant, which had an internal time of CT16, for 1 hour (Fig. 3A,B). CT16 moths showed higher foraging activity than CT4 moths (Fig. 3C). Approximately ~63% of CT16 moths visited flowers in the first 10-minute segment of the experiment, while only 10% of CT4 moths did in the same time window (t-test; p-value = 0.004). While CT16 moths showed greater activity than CT4 moths throughout the hour, CT16 moth activity did taper, with 23% of CT16 moths contacting flowers during the last 10-minute segment. These results suggest that even in the presence of a scent emitting plant, if a moth’s internal biological time is the morning, the moths do not respond effectively to foraging cues.

Altogether, these results indicate that having both organisms’ circadian clock in a certain phase is important for initiating their interaction.

### *M. sexta* locomotor activity is clock regulated but also light dependent

Generally speaking, aspects of physiology and behavior that recur at certain times of day could be regulated by daily light/dark conditions, the internal circadian clock, or interplay between both. To examine whether the *M. sexta* circadian clock influences daily moth locomotor activity, the locomotor activities of naïve male moths, which were entrained to 12-hour light/12-hour dark conditions (12L:12D), were analyzed under a variety of light conditions.

**Figure 4:**
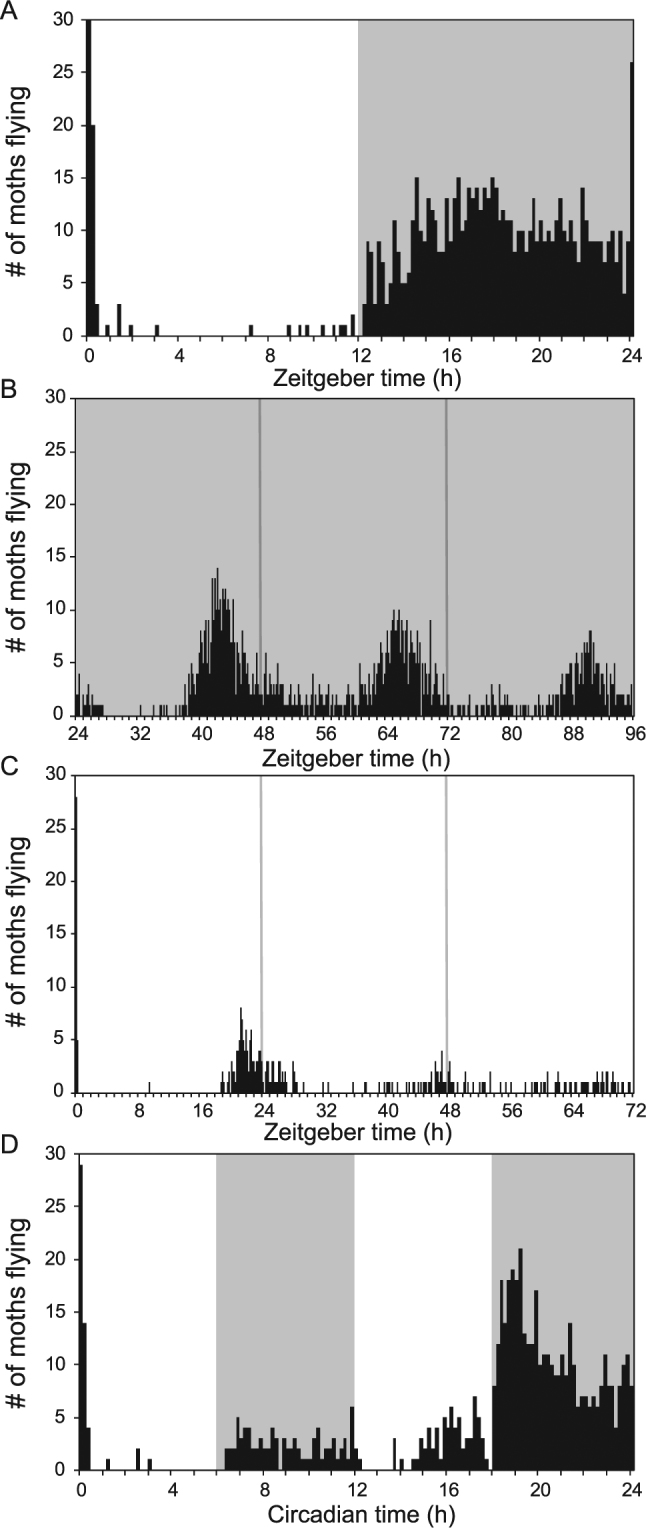
*M. sexta* activity is clock regulated but light repressed. Actograms of the number of male *M. sexta* moths flying in each 10-minute window of 24 hours or 72 hours. Experiments were repeated independently with 15 male moths three times. The results show the accumulated counts of flying moths in each time window. (**A**) 12L:12D. (**B**) Continuous dark for 3 days (3D DD). (**C**) Continuous light for 3 days (3D LL). (**D**) 12-hour T-cycle.

In 12L:12D conditions, moths begin to fly shortly after lights-off at Zeitgeber Time 12 (ZT12), increasing in activity until a peak around the middle of the night, after which activity begins to decline until the end of night (ZT24) (Fig. 4A). “Zeitgeber Time (ZT)” refers to the time (in hours) past the stimulus which resets the clock: in this case, light at dawn (dawn being ZT0). Around ZT0, a brief but significant spurt of activity occurs immediately after the overhead lights illuminate. In continuous dark (DD) conditions, moths exhibit oscillatory flight behavior during subjective night for at least three days, confirming the role of an endogenous circadian mechanism for locomotor activity (Fig. 4B). “Subjective night” refers to the nocturnal phase of circadian time (or CT) that the organism’s internal clock is currently experiencing, regardless of the actual time, light conditions, etc. In continuous light (LL) conditions, flight activity was severely dampened throughout the experiment, implying that light represses general locomotor activity in *M. sexta* (Fig. 4C). To highlight the interplay of the circadian clock and light conditions in determining locomotor activity in *M. sexta*, a 12-hour T-cycle (which means one light-dark or day-night cycle occurs in 12 hours, instead of 24 hours) was performed (Fig. 4D). A T-cycle is useful for extracting the separate roles of clock and light inputs to rhythmic behaviors. If a behavior is only light regulated, then one can predict high activity to occur either during light or dark periods (depending on the behavior). In this case, one would expect to see the same responses between the first and second 6-hour light (or dark) periods within the 24 hours. If a behavior is solely clock-regulated, one can expect rhythmic behavior to ignore light-dark transitions for the 24 hours of the T-cycle experiment. If a behavior is light-dependent and clock-regulated, activity is predicted to occur when the right light conditions align with the appropriate phase of time. During the hours corresponding with subjective day (CT0-12) in our T-cycle experiment, the moth flight activity was low across the different light conditions. A low period of activity is also maintained throughout the first half of subjective night (CT12-18), when the lights are on. In the second half of subjective night (CT18-24), a peak of activity occurs immediately after lights out, which gradually declines until dawn, closely mirroring the activity shown in 12L:12D and DD conditions for these timepoints. Thus, *M. sexta* flight activity is regulated by the interplay between external light conditions and internal circadian clock mechanisms. Taken together, these actograms provide evidence of strong clock regulation of the *M. sexta* locomotor, with additional suppressive regulation from light input.

### The circadian clock gates the sensitivity and behavioral response of *M. sexta* to floral odor signals

**Figure 5:**
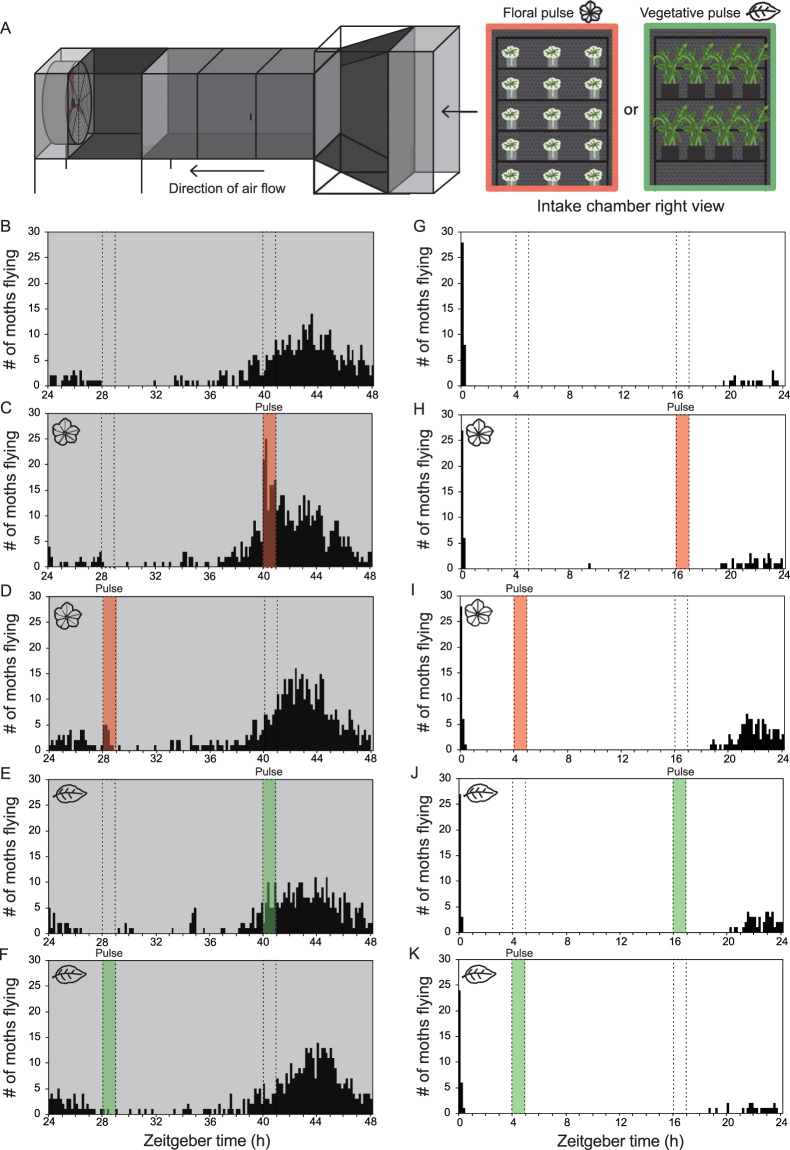
The circadian clock and light modulate behavioral response to floral scent. (**A**) The wind tunnel used for scent pulse experiments with diagrams of the floral and vegetative scent pulse setups administered to the intake of the wind tunnel. All floral scent pulses were administered by adding 15 cut flowers entrained to a night timepoint, ZT16. All vegetative scent pulses consisted of 8 two-month-old plants with no flowers. (**B–H**) Actograms of flight activity with subjective day/night stimulation pulses of floral scent or vegetative scent emissions. Each experiment consisted of 15 male moths entrained to 12L:12D and was repeated independently three times. The dotted lines indicate the time windows when either night or day scent pulses were given. (**B**) DD actogram (**C**) DD actogram with ZT40-41 “floral night pulse” (highlighted in pink). (**D**) DD actogram with ZT28-29 “floral day pulse”. (**E**) DD actogram with ZT40-41 “vegetative scent pulse” (highlighted in green). (**F**) DD actogram with ZT28-29 “vegetative scent pulse”. (**G**) LL actogram (**H***)* LL actogram with ZT16-17 “floral scent pulse”. (**I**) LL actogram with ZT4-5 “floral scent pulse”. (**J**) LL actogram with ZT16-17 “vegetative scent pulse”. (**K**) LL actogram with ZT4-5 “vegetative scent pulse”.

While moths exhibit circadian clock-regulated nocturnal activity in their locomotion (Fig. 4), can their behavior be altered by the presence of floral odors? To test this, we recorded moth activity in DD conditions and administered a “pulse” of floral scent by placing cut and visually-hidden *P. axillaris* flowers in the intake of the wind tunnel during the subjective night (ZT40) of the 2^nd^ day in DD for 1 hour (Fig. 5A–C). A significant increase in moth flight activity over unprovoked moths (average of 24 more moth flights per trial) occurred during this hour, indicating that moths will increase locomotor activity in search of unseen but smelled flowers (Fig. 5B,C). Is this response to floral presence also time dependent? “Gating” is a term used to describe the ability of the circadian clock to change sensitivity for a specific stimulus to a certain time window or “gate” of time. To determine if the circadian clock gates the response of *M. sexta* to floral odors, the effect of day and night-time scent pulses on moth flight activity was analyzed more fully by also administering a pulse in the subjective morning (ZT28) of the 2^nd^ day in DD. When naïve moths were exposed to a floral scent pulse at ZT28 in DD (Fig. 5D), a significantly smaller increase in moth activity appeared than that seen for the “night” floral scent pulse given at ZT40 [t-test of flights during the one-hour pulse at ZT28 (minus background) versus flights during the one-hour pulse at ZT40 (minus background), two-tailed, p-value = 0.029]. Floral scent pulses at ZT4 and ZT16 contributed no measurable increase in activity in the first day of LL conditions (Fig. 5H,I), providing further evidence that hawkmoth activity is strongly light repressed. To determine if scent from the *P. axillaris* vegetation could also affect male moth behavior, we administered a “vegetative scent pulse” by placing 8 two-month-old, non-flowering plants in the intake of the wind tunnel at the same day and night timepoints for the floral scent pulse experiments (Fig. 5). When the vegetative scent pulse was introduced at subjective morning and night timepoints, no deviation from unscented activity was observed in males (Fig. 5E,F,J,K), indicating that, at least in male *M. sexta* adults, vegetative scent does not elicit foraging behavior.

**Figure 6:**
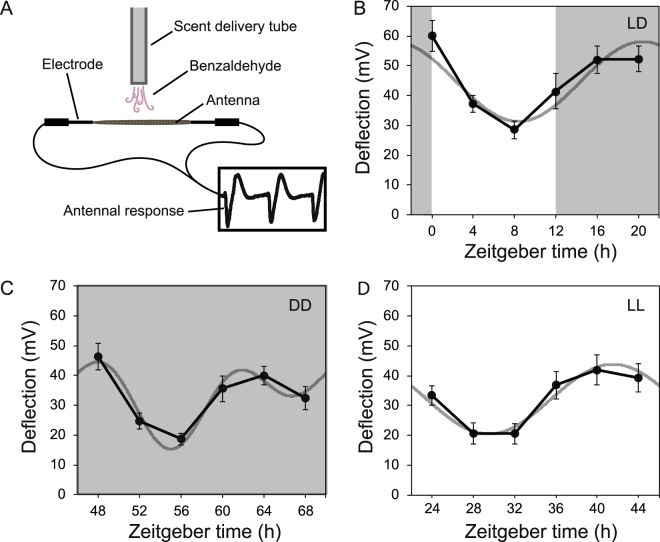
*M. sexta* exhibits a circadian rhythm in antennal responsiveness to the floral volatile benzaldehyde, regardless of light conditions. (**A**) Illustration of electroantennogram (EAG) setup (**B**) Benzaldehyde EAG in 12L:12D. (**C**) Benzaldehyde EAG in DD. (**D**) Benzaldehyde EAG in LL. The results are means ± s.e.m. (n = 12 for each timepoint). Fitted curves in the background are derived from cosinor analysis in CircWave. CircWave analysis for LD curved fit: F-stat = 12.61, p-value = 0.000024, R^2^ = 0.2795. CircWave analysis for DD curved fit: F-stat = 9.42, p-value = 0.000004, R^2^ = 0.3598. CircWave analysis for LL curved fit: F-stat = 12, p-value = 0.000034, R^2^ = 0.2581.

Given that moths exhibited a time-dependent behavioral response to floral scents, we hypothesized that this behavior could be partially caused by changing sensitivity/responsiveness in chemoreception to floral odors. Electroantennograms (EAG) have traditionally been used to examine the strength of the antennal (olfactory) sensitivity and responsiveness in insects^30^. To test whether antennal responsiveness to floral scent changed throughout the day and also whether this is regulated by the circadian clock, EAG responses of moth antennae to *P. axillaris* odorants were measured in 24-hour time courses (Fig. 6A). In 12L:12D conditions, based on statistical cosinor analysis (CircWave: https://www.euclock.org/results/item/circ-wave.html), our EAG time course results showed a significant fit to a cosine curve that oscillates over 24 hours, with a minimum response in the day separating periods of maximum response during the night (Fig. 6B). The timing of the higher EAG response coincides with the timing of moth foraging behavior. Experiments in free running conditions (DD and LL) showed similar rhythms (though both are slightly reduced in overall amplitude from LD) (Fig. 6B–D), indicating that the observed rhythms may result from an endogenous circadian-clock regulated mechanism.

**Figure 7:**
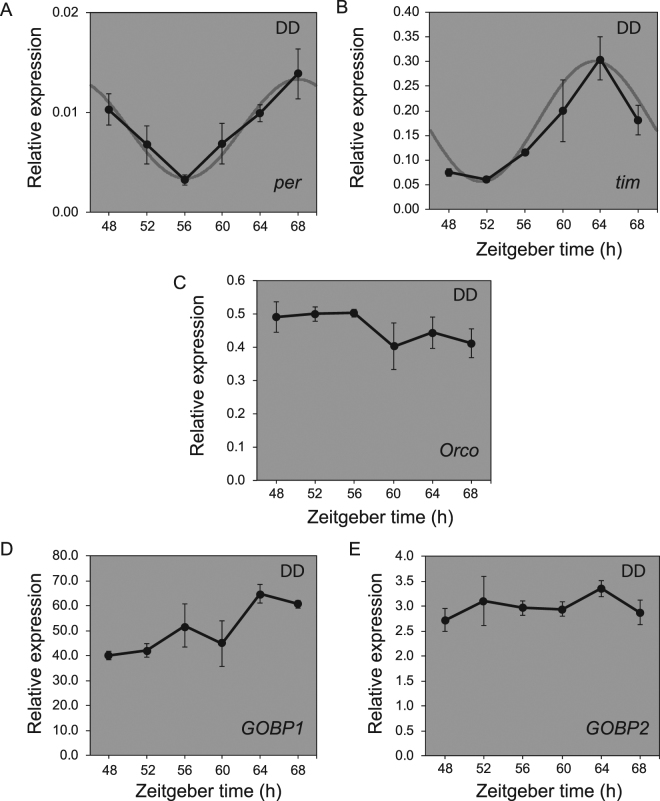
Circadian gene expression analysis in *Manduca sexta* antenna. qPCR data of (**A**) *per*, (**B**) *tim*, (**C**) *Orco*, (**D**) *GOBP1*, and (**E**) *GOBP2* gene expression profiles in continuous dark conditions. The results of each timepoint are relative values to the expression levels of control gene *rps13*. The results show means ± s.e.m. derived from three biologically independent experiments. Fitted curves in the background of A and B derived from cosinor analysis in CircWave. CircWave analysis for *per* curved fit: F-stat = 12.81, p-value = 0.000569, R^2^ = 0.6307. CircWave analysis for *tim* curved fit: F-stat = 17.8, p-value = 0.000109, R^2^ = 0.7036. CircWave was not able to find sine waves for Orco, GOBP1, and GOBP2.

As our results indicated the presence of a circadian rhythm of scent responsiveness in *M. sexta* antennae, we further examined what part of the odorant reception mechanism could be potentially involved in this oscillatory change in sensitivity. Recent work has sought to uncover the molecular mechanisms regulating odorant reception in insect antennae^31^. In brief, primarily hydrophobic odorants enter pore tubules of the sensillum and bind to odorant-binding proteins (OBPs) in order to traverse the aqueous sensillar lymph to odorant receptors (ORs). There seems to be a large diversity of OBPs and ORs across each insect species, with few highly-conserved homologs. One exception is odorant receptor coreceptor (Orco), a protein originally identified as a traditional OR that has been shown to act as a general coreceptor for other ORs, and is highly conserved among insects^32^. An examination of gene expression in moth antennae during continuous dark conditions (where EAG daily rhythms continued) showed circadian rhythmic expression of two likely moth clock genes, *period* (*per*) and *timeless* (*tim*) (Fig. 7A,B), indicating that the molecular clock is running in the moth antennae in DD. Under the same conditions, we did not observe obvious circadian oscillation of *Orco* gene expression (Fig. 7C), indicating that a general suppression of odorant reception does not occur through clock modulation of *Orco* expression. We also examined the temporal expression profiles of genes encoding two general odorant binding proteins, General Odorant Binding Protein 1 (GOBP1) and General Odorant Binding Protein 2 (GOBP2), which show some conservation in insects^33^. We did not observe the daily oscillatory patterns in their expression (Fig. 7D,E). These results indicate that the clock-dependent changes in floral odor sensitivity cannot explained by the daily expression patterns of these three olfactory genes.

## Discussion

### Clock synchrony is important to plant-pollinator interactions

Over the past several decades, knowledge of circadian clocks and their importance to biology has advanced significantly. The clock regulation of physiology and behavior appears to be robust: a famous example is found in *Arabidopsis thaliana*, where one third of mRNA transcripts are known to be under clock control^34^. Clocks are thought to be highly conserved and physiologically integrated in order to allow organisms to prepare for situations which can be readily predicted to occur in the future of the organism, giving them a “leg up” on the competition.

Given that many pollinators have temporal rhythms in their activity and can associate with many plants which also exhibit rhythmic attractive qualities^35,36^, we were interested in whether a shift in the synchronization of the plant-pollinator clocks would result in decreased attractiveness to moths. We found that changing the phase of the clock, either through entrainment or transgenic manipulation, did generally result in decreased moth preference for those plants (Figs 1 and 2). A recent study also showed that tobacco plants with altered circadian clocks had changes to their fitness, as measured by seed production as a result of outcrossing^29^. Circadian clock status in moths also influences foraging activity (Fig. 3). These observations indicate that both plants and insects time their mutual interaction

Co-evolutionary relationships between plants and pollinators can likely be modified or swapped for entirely new relationships based on phenotypic changes in any of the involved species. For plants, these phenotypic changes could include flower shapes, colors, scent composition, nectar volume; while in pollinators these could include chemosensory and visual capabilities, metabolic rates, mouth part morphology, etc. Many of the above examples are modulated by circadian rhythms, and so it is important to consider modification to clock regulation (or the clock itself) as an important factor in the establishment and disestablishment of plant-pollinator interactions.

### Moth behavior is clock regulated but also light dependent

Moth locomotor activity is clearly driven by the circadian clock, as is evident in continuous dark conditions (Fig. 4B). However, in all continuous light experiments, flight behavior was severely reduced throughout the time course, indicating that moths avoid flight in high light conditions regardless of the clock’s phase. This repression in behavior due to continuous light has also been observed in other insects, including *Drosophila melanogaster*^37,38^ and *Calliphora vicina*^39^. In *C. vicina*, the clock itself maintains rhythmicity, indicating that the constant dampening of locomotor activity occurs through means other than the clock^39^. The small amount of activity that was seen in *M. sexta* males occurred in the last few hours of the first day (Fig. 4C), which is delayed from the peak activity time in LD or DD conditions. This indicates that clock pace could also be slowed in LL conditions, a phenomenon seen in other insects during continuous light of relatively low intensity^37–39^.

In all experiments where moths exit a period of darkness and receive a sudden onset of light, moths exhibit a brief but large increase in flight activity (Fig. 4A). This effect seems to be much greater at ZT0 than at ZT12 (Fig. 4D). This dark-to-light transition behavior can also be observed in other nocturnal insects, such as bed bugs^40^. This behavior potentially exemplifies an escape response caused by a sudden increase in light, a situation which would occur when insect hiding places/harborages are exposed.

Previously, similar behavioral actograms of *M. sexta* (using long-day conditions, instead of 12L:12D conditions) were published, and also exhibited light-repressed circadian locomotor activity^41^. Taken together, our study supports the notion that the daily flight activity of *M. sexta* is controlled by the interplay between external light conditions and internal circadian timing mechanisms.

### Behavioral response to floral scent is modulated by the circadian clock

*M. sexta* showed a greater response to floral odor stimuli at night in DD (Fig. 5B–D), indicating that the clock gates the ability of the moth to respond to odor. Temporal modulation of behavioral responses to odors were documented before in insects, though in non-floral settings, usually in response to pheromone or food-related odors. An early example of temporal modulation to pheromones can be found in *Epiphyas postvittana*, where male moths exhibited a strong response to female sex pheromones during the evening, with greatly reduced sensitivity during other portions of the day^42^. Food-related examples can be found in disease vectors including the tsetse fly, *Triatoma infestans*, and *Rhodnius prolixus*, which all show daily rhythmic responses to host odors^27,28,43^.

While no apparent response to vegetative tissue was shown in any of the scent pulse experiments (Fig. 5E,F,J,K), it is important to note that all moths in this study were male. Female moths of *M. sexta* are well known to oviposit on Solanaceous host plants, like *Solanum*, *Nicotiana*, and also *Petunia*^44^. It is thus possible that female moths would show an increase in flight activity in response to emissions from vegetative tissue. Whether this response would also be clock-regulated is another question. Female moths of *Heliothis virescens* do show differential oviposition responses to herbivore-induced volatiles from vegetative tissues collected from day and night timepoints^45^.

To further investigate a potential mechanism of the time-dependent response to floral odors, we examined *M. sexta* olfactory sensitivity in a series of time courses. Indeed, olfactory neurons in moth antennae showed a clear 24 h oscillation of responsiveness in 12L:12D, LL, and DD conditions (Fig. 6). While a novel finding in plant-pollinator interactions, circadian modulation of antennal sensitivity was documented in other species. In *D. melanogaster*, flies showed increased sensitivity to ethyl acetate, an attractive compound, as well as to benzaldehyde (which, in contrast to *M. sexta*, *D. melanogaster* avoids)^24^. In addition, in the disease vector *Anopheles gambiae*, temporal changes in sensitivity to host odors was described^46^.

A key finding in understanding circadian rhythmicity in antennal responses was shown in *D. melanogaster*, where rhythmic EAG responses were observed in flies lacking the nerve cells containing the central oscillators^26^. A targeted reduction of clock gene expression in antennal tissues abolished the EAG rhythms, indicating that peripheral clocks in the antenna are responsible for regulating rhythmicity in olfactory sensitivity^26^. At the same time, a study in cockroaches found that severing the optic nerve also abolished rhythmicity in EAG recordings, indicating that the central input can affect olfactory sensitivity^47^. While we show that the antennal clock in *M. sexta* is likely oscillating (Fig. 7A,B), the transcriptional levels of conserved components of the odorant reception machinery that we tested (*Orco*, *GOBP1*, and *GOBP2*; Fig. 7C–E) did not show rhythmic patterns in gene expression. This contrasts with experiments in mosquitos (*A. gambiae*) showing rhythmicity in both *OBP* RNA and protein abundance^46^, as well as *Orco* mRNA^48^. Currently, we cannot rule out the possibility that other genes involved in *M. sexta* odorant reception show circadian oscillation. Further examination of expression of other Odorant Binding Proteins, Odorant Receptors, and signaling components will be necessary to elucidate the mechanism behind rhythmic EAG responses in *M. sexta*.

Beyond circadian sensitivity, however, it is possible that other factors also contribute to the time-dependent behavioral response seen in Fig. 4, including general locomotor activity. Oscillation of EAG response continues in LL conditions (Fig. 6D), indicating the lack of response to ZT16 floral scent pulse in LL conditions (Fig. 5H) is likely due to impairment of locomotor activity.

To fully understand the insect’s temporal response to odors, additional studies must further explore the mechanisms by which the central and antennal clocks interact with olfaction machinery in the antennae. While work in this field will be much easier in *D. melanogaster*, applying knowledge gained from fruit flies to important pollinator species will allow for insight into how plant-pollinator interactions are tuned by their circadian clocks.

## Methods

### Plant cultivation and insect rearing

*Petunia* plants (*Petunia axillaris* and *Petunia hybrida*, cv. Mitchell) were grown in nutrient-enriched potting soil (Sunshine 4 Mix, Sun Gro Horticulture) in a growth room at 25 °C and under 12L:12D conditions. Light from full spectrum fluorescent lamps (Octran F032/950/48, Osram-Sylvania) was set to an approximate fluence rate of 100 µmol/m^2^/s. Generation of all transgenic lines was described previously^23^.

To avoid mating and oviposition behaviors, all experimental *M. sexta* moths were male. Moths were obtained from a rearing facility in the Department of Biology at the University of Washington, Seattle, USA. Larval diet was described previously^49^. Pupa were entrained and allowed to eclose in a 12L:12D light cycle, 25 °C, 65% humidity. Once eclosed, adult moths used for experiments were kept flower-naïve to eliminate learned behaviors and food-deprived to eliminate any unforeseen (and untested) food-related impacts on the circadian clock.

### Actograms and behavioral assays

All behavior experiments were conducted in a plexiglass wind tunnel (see Supplementary Fig. 1), dimensions of 2.5 × 1 × 1 m^3^ (L × W × H). Airflow was set to 0.1 m/s. Overhead fluorescent lights were used to simulate daylight, at 100 µmol/m^2^/s. Night light (dark period) was simulated by turning off the overhead lights and turning on an indirect light source from 50 ft away. Photon density during dark periods was too low to measure using our photometer (LI-250A, LI-COR), and even though it was not possible for examiners to see chamber activity without the aid of infrared lighting and cameras, moths seemed to be able to navigate quite readily.

Prior to the experiments, 15 adult male *M. sexta* were introduced to the chamber 1 day early and entrained to the 12L:12D light cycle. During recording, the number of moths flying in every 10-minute period was counted. During dark periods, infrared lighting was used in tandem with a camera (Basler Pilot GigE, Basler Vision Technologies) to visualize movement. Infrared lighting was kept on for all light periods. Each experiment was repeated at least 3 times.

For behavioral choice assays, naïve male moths obtained from the rearing facility were entrained to a 12L:12D light cycle at 25 °C, 50% humidity in a Percival environmental chamber for 1 day after eclosion. Standard error of the mean (s.e.m.) was calculated as^50^:

$${\rm{s}}.{\rm{e}}.{\rm{m}}.={(\frac{p(1-p)}{n})}^{\frac{1}{2}}$$

where *p* is the observed proportion and *n* is the number of observations. Statistical analyses were performed in R (www.r-project.org) using the binomial exact test.

For the experiment in Fig. 3, each experimental trial utilized 20 male moths at a time (10 moths at CT4 and 10 moths at CT16), which were entrained in separate chambers. The experiments were repeated in full three times with independent sets of moths and plants. 30 minutes prior to the experiment (=ZT15.5), the 20 moths were transferred to the wind tunnel chamber to let them adjust to the new environment. The plant with flowers (internal time, CT16) was introduced at the beginning of the experiment. Infrared camera recording was started from ZT16 and ended at ZT17. For each 10-minute segment between ZT16 and ZT17, the number of individual moths that visited flowers was scored (in our scoring system, a “visit” requires the moth’s proboscis to physically make contact with the flower, and the same individual could not score twice in the same 10-minute period). CT4 and CT16 moths were visually distinguished by dots of white and black nail polish on the dorsal side of the thorax. This marking method did not interfere with moth locomotor activity, and is enough to distinguish two groups of moths during the recordings.

For the experiments in Fig. 5, prior to scent pulse assays, whole flowers (2–3 days in age, experiencing CT16) were cut from plants in the growth room, placed in a glass vial containing a 5% sucrose solution and carried to the wind tunnel room in a sealed container. At the appropriate timepoint, flowers (still in vials) were arranged in a regular pattern (5 down the height axis, 3 across the width axis). Squares of brown construction paper (also used to line the floor of the wind tunnel’s test chamber) were taped in front of the flowers to block visual availability of the flowers to moths in the wind tunnel and assist in dispersing the floral odors. After the timepoint, flowers were immediately replaced into the sealed containers and removed from the room.

### Electroantennogram (EAG) recording

Moths were reared and trained as mentioned above and placed in the wind tunnel for 1 additional day of entrainment prior to the experimental day. About 10 minutes prior to the timepoint, antennae were excised from moths and the basal end hydrated in electrode gel (Spectra 360, Parker Laboratories). For dark time points, antennae were harvested under dim red light and immediately transferred in dark containers to the EAG room, where EAG recordings took place in the dark. The antenna from a single male moth was connected to two glass-electrodes filled with conductive gel. The EAG signal was recorded by Ag-AgCl wires connected to the headstage of an extracellular amplifier (1800, A-M Systems, Sequim, WA), to achieve 100× amplification, and collected using WinEDR acquisition software (WinEDR v3.5; University of Strathclyde, Glasgow). The signal was filtered and digitized at 400 Hz sampling rate. Olfactory stimuli were delivered to the antenna by pulses of air from a constant air stream diverted through a glass syringe containing a piece of filter paper bearing the odor stimuli. The odor pulses were injected into a charcoal-filtered air stream flowing to the side of the antenna at a rate of 100 ml/min. The stimulus was pulsed by means of a solenoid-activated valve controlled by the WinDaq acquisition software. Odor syringes containing 10 µL of a 1:100 dilution of synthetic odorants diluted in mineral oil were prepared. Scent pulses were delivered every 20 seconds for 5 total pulses (see Supplementary Fig. 2). EAG amplitudes (mV) were measured for each odor pulse, and the average of the 5 pulses was recorded as a datapoint. Each timepoint represents an accumulation of data from 12 individual antennae from as many moths. The statistical cosinor analysis was performed with EAG recording data using CircWave software (https://www.euclock.org/results/item/circ-wave.html) to assess the potential circadian rhythmicity of EAG time course results.

### Gene expression analysis

Male moths (*M. sexta*) entrained post eclosure in 12L:12D cycle were introduced to the wind tunnel chamber 1 day prior to the experiment. At each timepoint, all moth tissues were immediately flash frozen in liquid N_2_. While still frozen, individual tissues were dissected and separated into 2 mL tubes with 2 steel beads. Tissue was ground in tubes while frozen (Mixer Mill MM 400, Retsch Technology). Total RNA was extracted by TRIzol-based method and received DNAse treatment. cDNA synthesis and qPCR analyses were performed as described previously^51^. The statistical cosinor analysis was performed with qPCR time course results using CircWave software (https://www.euclock.org/results/item/circ-wave.html). Primers for qPCR as follows (per primers from Schuckel *et al*.^52^): RPS13-F 5′-GTCTTGCCCCTGACCTACCT-3′, and RPS13-R 5′-TGGCAGCACACTCTTTGTCT-3′ for the *rps13* gene (internal control); PER-F 5′-CCGCATCCGCCGCTACC-3′, and PER-R 5′-TGCAATCATGGCGGTGAAC-3′ for *per*; TIM-F 5′-GCTGCTCAGGAATATCTTGCAT-3′, and TIM-R 5′-GGATCTGGTTTTGTACGGTGTG-3′ for *tim*; ORCO-F 5′-ACAGCCACCCACCCATTGTTCACG-3′, and ORCO-R 5′-GGTCTCGTTCGTCTCCTTGTT-3′ for *Orco*; GOBP1-F 5′- GCCACTTCAACCTGCTCACC -3′, and GOBP1-R 5′- GGTCCTCTTCTGCGTCGTGT -3′ for *GOBP1*; GOBP2-F 5′-ACACGCATCCATCACGTCAA-3′, and GOBP2-R 5′-CGTCGTATTGCTTCTCGCAGT-3′ for *GOBP2*.

## Electronic supplementary material

Supplementary figures
