# Supplementary material for: Circadian clocks of both plants and pollinators influence flower seeking behavior of the pollinator hawkmoth Manduca sexta
Source: Sci Rep. 2018 Feb 12;8:2842. doi: 10.1038/s41598-018-21251-x (PMC5809389; doi:10.1038/s41598-018-21251-x)
Supplement: Supplementary file 1 — Supplementary figures [file 41598_2018_21251_MOESM1_ESM.doc]

**Supplementary Information**

**Circadian clocks of both plants and pollinators influence flower seeking behavior of the pollinator hawkmoth *Manduca sexta***

Myles P. Fenske, LeAnn P. Nguyen, Erin K. Horn, Jeffrey A. Riffell, and *Takato Imaizumi

Department of Biology, University of Washington, 24 Kincaid Hall, Box 351800, Seattle, WA, 98195-1800, USA

*Corresponding, [takato@u.washington.edu](mailto:takato@u.washington.edu)


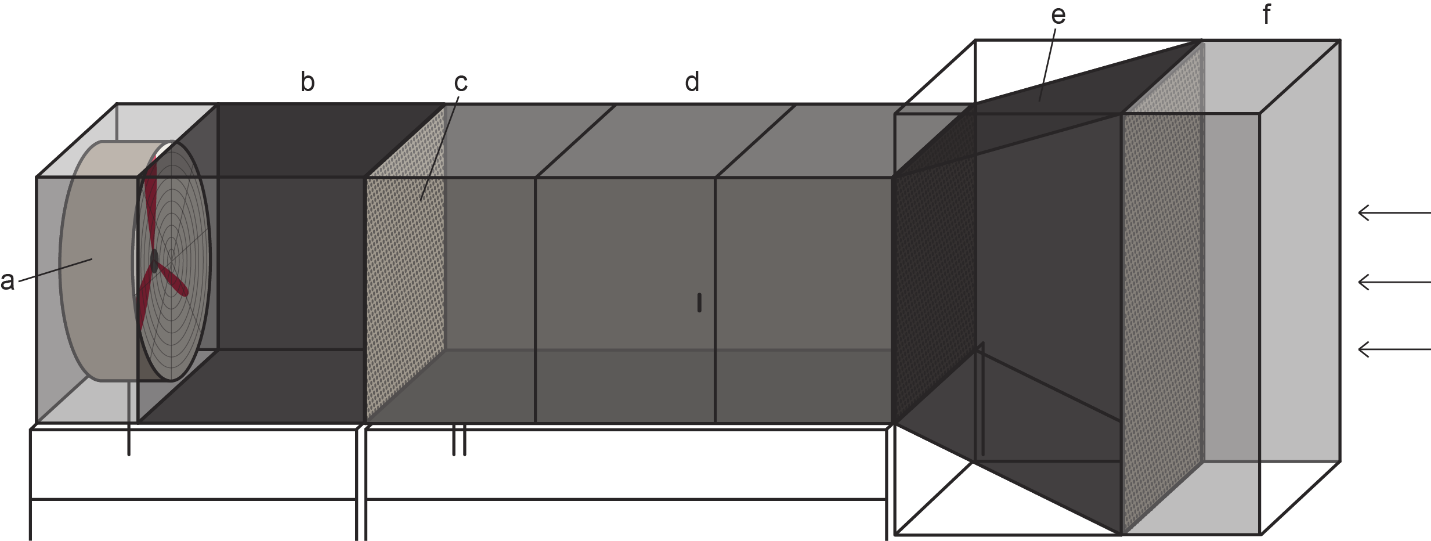


**Supplementary Figure 1.** Diagram of the wind tunnel apparatus used for all behavioral experiments. (*a*) Fan. (*b*) Diffuser. (*c*) Flow straightener. (*d*) Experimental chamber, dimensions of 2.5x1x1 m^3^, LxWxH. (*e*) Contraction section. (*f*) Intake and flow straightener. The direction of air flow is indicated by arrows.


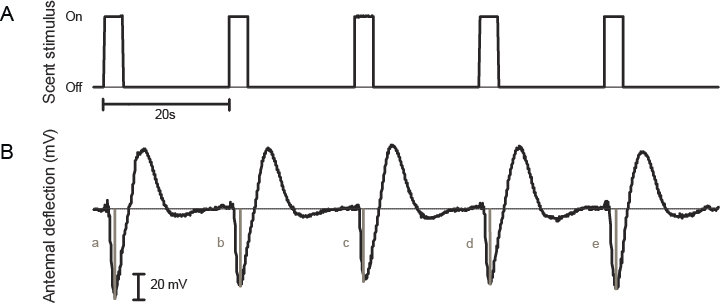


**Supplementary Figure 2.** Diagram of antennogram protocol. (*A*) For each electroantennogram datapoint, a detached antenna is subjected to a series of 5 brief scent pulses every 20 seconds. (*B*) The maximal deflection amplitude is recorded for each of the 5 scent pulses (a-e). Each data point is the mean of those 5 recorded deflection values (n = 12).
